# Workflow efficiencies for flexible cystoscopy: comparing single-use vs reusable cystoscopes

**DOI:** 10.1186/s12894-024-01436-5

**Published:** 2024-03-06

**Authors:** Ian Haislip, Dinah Rindorf, Christina Cool, Brittany Tester

**Affiliations:** 1 Ambu USA, Health Economist, 6271 Columbia Gateway Drive, Suite 200, Columbia, MD, 21046 USA; 2 Ambu A/S, Ballerup, Denmark; 3 Regional Urology, Shreveport, LA USA; 4 Ambu USA, Columbia, MD USA

**Keywords:** Cystoscopy, Disposable equipment, Endoscopy, Organizational efficiency

## Abstract

**Background:**

Flexible cystoscopy is a common procedure to diagnose and treat lower urinary tract conditions. Single-use cystoscopes have been introduced to eliminate time-consuming reprocessing and costly repairs. We compared the hands-on labor time differences between flexible reusable cystoscopes versus Ambu’s aScope™ 4 Cysto (aS4C) at a large urology Ambulatory Surgery Center (ASC).

**Methods:**

Reusable and single-use cystoscopy procedures were shadowed for timestamp collection for setup and breakdown. A subset of reusable cystoscopes were followed through the reprocessing cycle. T-tests were calculated to measure the significance between groups.

**Results:**

The average hands-on time necessary for reusable cystoscope preparation, breakdown, and pre-cleaning was 4′53″. Of this, 2′53″ were required for preparation, while 2′0″ were required for breakdown and pre-cleaning. The average hands-on time for reprocessing for reusable was 7’1” per cycle. The total time for single-use scopes was 2′22″. Of this, 1′36″ was needed for single-use preparation, and 45 s for breakdown. Compared to reusable cystoscopes, single-use cystoscopes significantly reduced pre and post-procedure hands-on labor time by 2’31”, or 48%. When including reprocessing, total hands-on time was 80% greater for reusable than single-use cystoscopes.

**Conclusion:**

Single-use cystoscopes significantly reduced hands-on labor time compared to reusable cystoscopes. On average, the facility saw a reduction of 2′31″ per cystoscope for each procedure. This translates to 20 additional minutes gained per day, based on an 8 procedures per day. Utilizing single-use cystoscopes enabled the facility to reduce patient wait times, decrease turnaround times, and free up staff time.

## Background

Flexible cystoscopy is a common urologic procedure performed in health care facilities to treat and diagnose conditions in the lower urinary tract. Traditional reusable flexible cystoscopes must undergo numerous steps to ensure the device is reprocessed and ready for each procedure. These steps include precleaning, leak testing, cleaning, disinfection, rinsing, drying and storage [1]. Each step requires significant hands-on labor from staff members at the facility where the procedure is taking place. The numerous steps are vulnerable to process fluctuations which may cause delays or cancellations if the cystoscopes fail to be prepared in time for each procedure [2, 3]. To mitigate this risk, facilities may take preventative measures such as increasing allocated appointment times whenever daily procedure volumes exceed the number of reusable cystoscopes.

In recent years, single-use endoscopes have become increasingly popular in the endoscopic market. Single-use endoscopes are used for one procedure only and then discarded. Therefore, these endoscopes are sterile immediately out of the package, and do not require numerous reprocessing steps or monitoring (e.g. routine micro-biological tests or visual inspection). A disposable cystoscope released to the market in 2020 has shown satisfactory clinical performance compared to reusable cystoscopes across multiple studies [4, 5]. By eliminating numerous reprocessing steps, facilities may be able to reduce hands-on labor from staff-members by implementing a single-use cystoscope into clinical practice. Furthermore, a facility may be able to increase daily patient throughput or complete other necessary administrative tasks more efficiently with conversion to single-use cystoscopes [6, 7]. Therefore, this study aimed to identify the hands-on labor time differences observed at a large urology Ambulatory Surgery Center (ASC) after converting to single-use cystoscopes.

## Methods

### Process mapping

**Fig. 1:**
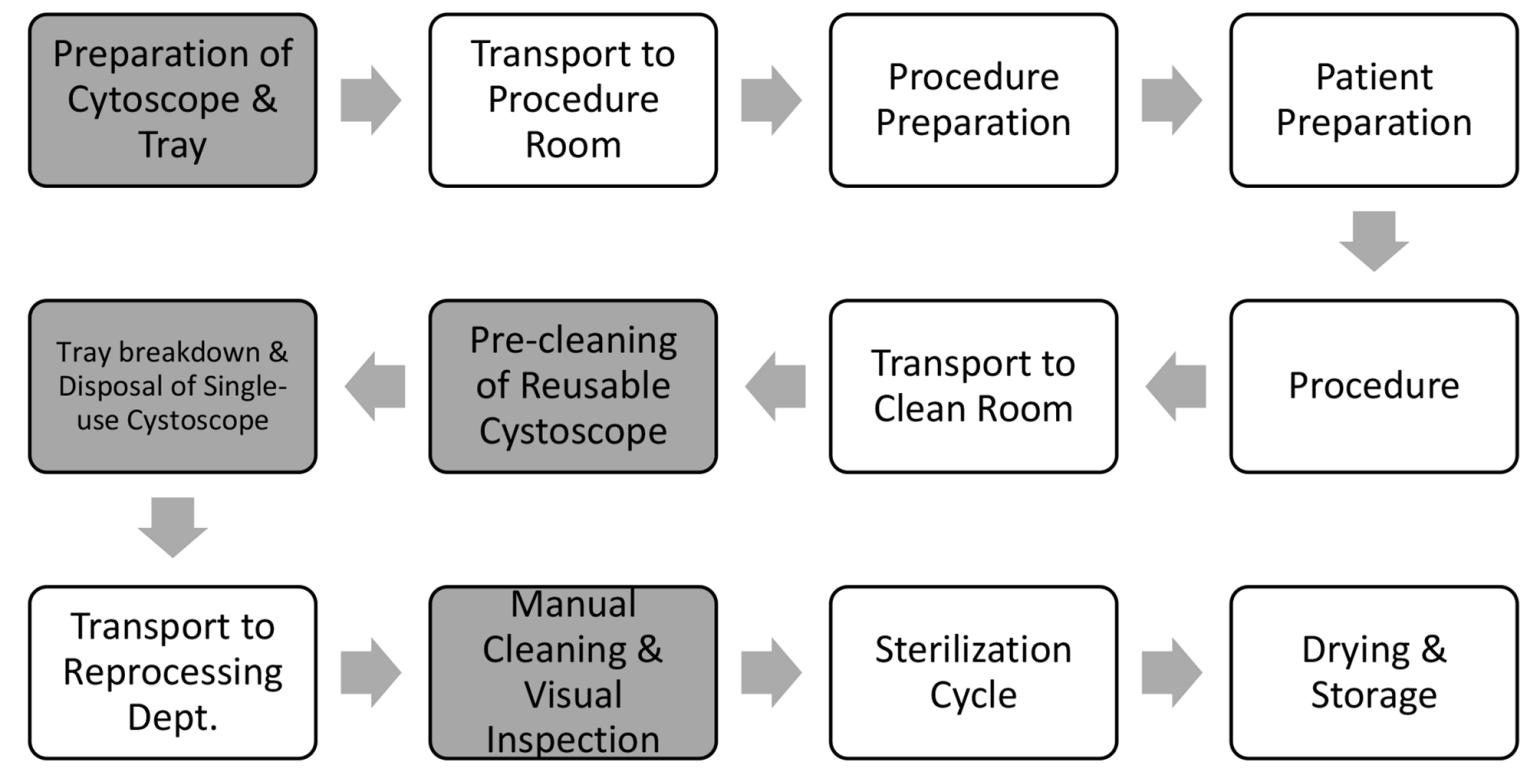
Procedure flowchart that breaks down each step in a cystoscopy procedure, with distinct steps for preparing and reprocessing cystoscopes before and after procedures. Boxes shaded gray denote where in the process timestamps were collected for this study

Figure 1 below outlines the different distinct steps for preparing and reprocessing cystoscopes before and after cystoscopy procedures at a large ASC in the United States.

Steps with definitions and associated volumes are as follows: The first step of the process was identified as Preparation of Cystoscope & Tray (Reusable, RU = 31, Single-use, SU = 20). In this step, trays were prepared with Operating Room (OR) drapes and towels, a sterilized reusable or single-use cystoscope, and the appropriate tool dependent upon the procedure type that day (i.e. graspers, BOTOX® needle, etc.). The cystoscopes and trays were prepared in bulk in contrast to preparing cystoscopes and trays directly preceding a procedure. After this step, the tray with equipment needed for the procedure was transported to the procedure room, and a nurse prepared the patient for the procedure by administering lidocaine and cleaning the patient. As a patient is brought back into the procedure room, the nurse also conducts typical administrative work for charting purposes, such as overall wellness questioning and patient identification.

After the procedure, the cystoscopes were transported to what the facility referred to as a ‘clean room’ where the cystoscope pre-cleaning and breakdown took place. Timestamps referred to as “Pre-cleaning of Reusable Scope” (RU = 20) and “Tray Breakdown & Disposal of Single-Use Scope” (SU = 20) consist of the manual pre-cleaning of reusable cystoscopes, cleaning of the transport tray, discarding the disposable materials (e.g. single-use cystoscopes), and placement of the reusable cystoscope into the transport container for sterilization. For single-use cystoscopes, what is referred to as “Tray Breakdown & Disposal of Single-Use Scope” (SU = 20) consists of discarding of the single-use cystoscope along with all disposable materials and cleaning of the tray. Although the following steps were not included in the total calculation of our time analysis, reusable cystoscope sterilization took place following the completion of cystoscope breakdown.

These steps included transport to reprocessing department, manual cleaning, visual inspection, sterilization, drying and storage of the reusable cystoscopes. The “Manual Cleaning & Visual Inspection” (RU = 4) process step captured timestamps around the hands-on labor required for the manual cleaning and visual inspection reprocessing steps for the reusable cystoscopes. For reusable cystoscopes, transport to the reprocessing department and drying cycle were not observed.

The automated sterilization step was not observed, as each cycle is pre-programmed, and did not involve hands on time. Following completion of the hands-on steps, reusable cystoscopes are placed into the low-heat sterilizer and ran through one pre-programmed cycle that lasts for 35 min. More information on timestamp collection can be found below in Sect. 2.2 “Data Collection”.

### Data collection

Data was collected between February 1–5, 2020 and March 23–25, 2020 at a large urology ASC in the US. The facility would utilize one type of cystoscope for all procedures on a given day. On occasion, some days had procedures using a reusable cystoscope and procedures using single-use cystoscopes. When this occurred, morning cystoscopies were performed with one cystoscope type with afternoon cystoscopies utilizing the other cystoscope type. The type of cystoscope used did not vary by the type of cystoscopy procedure being performed, hence both reusable and single-use cystoscopes were utilized for BOTOX injections, stent removals, and diagnostic procedures.

According to the process map shown in Fig. 1, boxes shaded gray denote where in the process timestamps were collected for this study. Preparation of Cystoscope & Tray timing began when the nurse broke the seal of the sterile glove packaging prior to dressing in the required personal protective equipment (PPE), which preceded the cystoscope and tray preparation and ended once the nurse disposed of the PPE. Following completion of procedures, timing of the Pre-cleaning of Reusable Cystoscope began once the nurse opened the door to the clean room to wheel in the tray carrying the contaminated cystoscope. The clean room was situated between procedure rooms and used for the manual cleaning of the reusable cystoscopes. Timing was concluded when the nurse conducted the final wipe down of the tray after discarding all other materials.

In addition to timing of Preparation of Cystoscope & Tray and Tray Breakdown & Disposal of Single-use Cystoscope, time stamp collection took place around the Manual Cleaning & Visual Inspection, and Sterilization Cycle for a subset of the reusable cystoscopes. The manual cleaning, cleaning verification, and sterilization cycles were followed and timed for four reusable cystoscopes. The facility did not collect cystoscopes for reprocessing immediately following each procedure, but instead collected and transported the cystoscopes periodically throughout the day. Timing began when the certified reprocessing technician picked up the first transport container containing a dirty reusable cystoscope. The remaining steps requiring hands-on labor were timed individually, including manual cleaning, visual inspection, and leak testing. Procedures were not timed, as duration of the procedure would not differ between the cystoscope types, as noted by the nurses and physicians.

### Statistical analysis

Upon completing the data collection for the procedures utilizing single-use and reusable cystoscopes, a two-sample t-test was conducted to determine whether the difference in cystoscope preparation and breakdown times was statistically significant between single-use and reusable cystoscopes. A two-sample t-test was performed using the software package Stata/SE version 16.1, StataCorp.

## Results

The average time per use needed for reusable cystoscope preparation, breakdown of the tray, and pre-cleaning of the cystoscope post-procedure amounted to 293.8 seconds (4’53”), with 173.7 s (2’53”) needed for Preparation of Cystoscope & Tray and 120.1 s (2’0”) needed for Pre-cleaning of Reusable Cystoscope and Cystoscope Breakdown. The average hands-on time observed for reprocessing reusable cystoscopes was 421.8 s (7’1”) and included a 35-minute automated sterilization cycle. Therefore, the total time for reprocessing excluding pre-cleaning was 2,521.8 s (42’1”).

When following the same protocols for procedure preparation with single-use cystoscopes, the total time needed for Preparation of Cystoscope & Tray and Tray Breakdown & Disposal of Single-use Cystoscope amounted to 142.1 seconds (2’22”). Preparation of Cystoscope & Tray for single-use cystoscopes time took 96.8 s (1’36”), followed by 45 s for Tray Breakdown & Disposal of Single-Use scope. Single-use cystoscopes saved the facility 151 s (2’31”) per cystoscope when compared to reusable cystoscopes.

When performing a two-sample t-Test assuming unequal variances for comparing the preparation times for reusable cystoscopes (*n* = 31) versus Ambu’s aScope 4 Cysto (*n* = 20), a significant difference in hands-on time between single-use and reusable cystoscopes for Preparation of Cystoscope & Tray was found (173.7s vs. 96.8s; *p* < .001). This analysis also found a significant relationship difference in hands-on time between single-use and reusable cystoscopes for Cystoscope Breakdown (120.1s vs. 45.3s; *p* < .001). Table 1 below summarizes the key findings for the cystoscope preparation and breakdown timestamps when using reusable and single-use cystoscopes.

### Reprocessing results

The average total time required for reprocessing the 4 reusable cystoscopes, which includes precleaning, leak testing, cleaning and rinsing, was 421.8 seconds, or 7’1”. Single-use cystoscopes do not require reprocessing and are disposed of immediately following the procedure, therefore saving this facility 7’1” in hands-on labor time for their reprocessing staff.

**Table 1:** Mean process times (s) and 95% Confidence Intervals (CI) for the steps Cystoscope & Tray Preparation, Cystoscope Breakdown and Cystoscope Reprocessing

Procedure Step	Reusable Mean (95% CI)	Single-use Mean (95% CI)	*P*-value
Cystoscope & Tray Preparation	173.7*s* (157.7-189.7)	96.8*s* (77.5–116.0)	< 0.001
Cystoscope Breakdown	120.1*s* (97.3-142.9)	45.3*s* (38.8–51.7)	< 0.001
Cystoscope Reprocessing	421.8s (240.5-603.1)	0.0*s*	0.003
**Total**	**715.6** ***s***	**142.1** ***s***	

## Discussion

Single-use cystoscopes proved to be more time efficient and saved, on average, 151 seconds (2’31”) per cystoscopy via pre-procedure preparation and post-procedure breakdown, excluding the time required for reprocessing. This figure translates to roughly 20 additional minutes gained per day, based on an 8 procedures per day volume. While this time may not seem significant and 20 min may not justify substantial differences made to clinical capacity or workflow, time savings are crucial for hospitals given backlog and staffing issues in today’s environment. This daily time savings could be utilized to increase patient throughput with a new procedure or shortening previous appointment block times by eliminating the need for reprocessing and recleaning of reusable cystoscopes. Additionally, the availability of single-use cystoscopes may enable facilities, such as this high-volume ASC, to add new cases on the same day without disrupting or delaying the scheduled workflow. This finding can be seen in a recent study by Medairos et al. where time savings attributable to switching from reusable to single-use cystoscopes allowed for an increase in patient throughput resulting in 9 additional patients being seen in a day (12 patients were seen with reusable cystoscopes versus 21 with single-use) [8]. Conversely, rather than increase procedure volumes, the time gained could be utilized for administrative tasks by nursing staff and administrators to improve workflow.

In addition to the previous study mentioned, additional recent studies have been interested in investigating the organizational impact of implementing single-use cystoscope into urology practice. Baston et al. aimed at investigating if the increased availability of single-use cystoscopes had an influence of stent dwell time and procedure cancellation rates. The study concluded that the implementation of single-use cystoscopes increased the clinical flexibility at their facility which helped decrease the stent dwell time, the readmission rate and procedure cancellation rate [9]. Similarly, a study by Phan et al. experienced a drop in the percentage of procedures subject to delays from 60% of patients, on average 6.4 days, compared to 0% of patients after implementing single-use cystoscopes [10].

While the evidence prior to this study and the time-savings presented above favor the use of single-use cystoscopes, there are other factors to consider before implementing single-use cystoscopes. An important element to consider is the cost ramifications associated with converting to single-use. A few studies have investigated the per procedure cost of reusable cystoscopes compared to single-use cystoscopes. In these studies, the cost per procedure for reusable cystoscopes ranges from $155–496 which showed that single-use in some cases may be cost-efficient when compared to cost of approximately $200 USD for per procedure for single-use cystoscopes [3, 11–14]. Several studies have investigated the cost-savings associated with single-use cystoscopes providing the ability of moving cystoscopy procedures from more costly settings, like operating and endoscopy rooms, to less costly settings like office and consultation rooms [6, 7, 15–17]. In these cases, all investigations find significant cost-savings of implementing single-use cystoscopes to their practice.

Finally, interest in the environmental impact of single-use endoscopy continues to grow as more facilities consider utilizing the disposable devices. Multiple publications examining the carbon footprint, total waste mass, and water consumption of single-use and reusable cystoscopes found single-use to be comparable or favorable in these metrics [18, 19]. In particular, one European hospital found that the use of single-use cystoscopes would reduce hard waste generation and water consumption by 946.8 kg per year and 94.68 m [3] per year, respectively [18]. A second investigation across 40 total cystoscopies found single-use cystoscopes generate less waste mass and total CO_2_ per case compared to reusable devices [19]. Given that the evidence available is limited, further studies are needed to assess the environmental impact of single-use vs. reusable cystoscopes.

### Limitations

Our study may have limitations that need to be considered when referencing the results. First, the site of our study took place at an Ambulatory Surgery Center that strictly focuses on urologic procedures and conditions; therefore, these results may not be generalizable. The day-to-day operations and preparation for procedures may vary significantly between sites and settings. For example, there were multiple staff members performing the reusable cystoscope pre-cleaning and reprocessing each day at this ASC, which is what we would expect to see in the real world. This can contribute to the variation in reprocessing duration but does not impact our comparison, as this is only impactful for reusable scopes as single-use cystoscopes are not reprocessed. Lastly, the latest reprocessing guidelines from societies [20, 21] have added new steps for adequate endoscope reprocessing, which would likely add significantly more time to reprocessing and sterilization cycles from what was observed during this study. In addition to the increased length of reprocessing cycles that follow the updated guidelines, facilities with fewer reusable cystoscope capital and high procedural demand would need to reprocess their scopes more frequently than facilities with higher capital inventory. Increased reprocessing turnaround time compounded with more frequent reprocessing means these facilities may see even greater time savings by utilizing a single-use cystoscope platform.

## Conclusion

Single-use cystoscopes require less hands-on time for pre-and post-procedure processes versus reusable cystoscopes and not only eliminate the need to reprocess the cystoscope post-procedure, but also the need for costly service contracts and repairs. The total scope of impact of single-use cystoscopes may present financial and operational enhancements for healthcare providers, while also creating favorable procedural processes for patients. Future studies should consider different care settings and organizational workflows to understand the full workflow impact of single-use cystoscopes.

## Data Availability

The datasets used and/or analyzed during this study are available from the corresponding author upon reasonable request.
